# EKF-Based Parameter Identification of Multi-Rotor Unmanned Aerial VehiclesModels

**DOI:** 10.3390/s19194174

**Published:** 2019-09-26

**Authors:** Rodrigo Munguía, Sarquis Urzua, Antoni Grau

**Affiliations:** 1 Department of Computer Science, CUCEI, University of Guadalajara, 44430 Guadalajara, Mexico; 2 Department of Mechanical Engineering, CUCEI, University of Guadalajara, 44430 Guadalajara, Mexico; isi.sarquis@gmail.com; 3 Department of Automatic Control, Technical University of Catalonia UPC, 08034 Barcelona, Spain; antoni.grau@upc.edu

**Keywords:** unmanned aerial vehicles, multi-rotor, parameter identification, observability analysis, Kalman filter

## Abstract

This work presents a method for estimating the model parameters of multi-rotor unmanned aerial vehicles by means of an extended Kalman filter. Different from test-bed based identification methods, the proposed approach estimates all the model parameters of a multi-rotor aerial vehicle, using a single online estimation process that integrates measurements that can be obtained directly from onboard sensors commonly available in this kind of UAV. In order to develop the proposed method, the observability property of the system is investigated by means of a nonlinear observability analysis. First, the dynamic models of three classes of multi-rotor aerial vehicles are presented. Then, in order to carry out the observability analysis, the state vector is augmented by considering the parameters to be identified as state variables with zero dynamics. From the analysis, the sets of measurements from which the model parameters can be estimated are derived. Furthermore, the necessary conditions that must be satisfied in order to obtain the observability results are given. An extensive set of computer simulations is carried out in order to validate the proposed method. According to the simulation results, it is feasible to estimate all the model parameters of a multi-rotor aerial vehicle in a single estimation process by means of an extended Kalman filter that is updated with measurements obtained directly from the onboard sensors. Furthermore, in order to better validate the proposed method, the model parameters of a custom-built quadrotor were estimated from actual flight log data. The experimental results show that the proposed method is suitable to be practically applied.

## 1. Introduction

Unmanned Aerial Vehicles (UAVs) with rotary wings (multi-rotors) allow great flexibility of movements, which makes them very useful for several tasks and applications. In this case, one of the main objectives of the research community has been the improvement of the autonomy of these systems. In this context, in order to develop autonomous control systems that perform well in terms of robustness, stability, precision, and adaptability, it is fundamental to have mathematical models that represent the actual dynamic behavior of the UAV in a precise manner.

Different methods have been proposed for deriving models of multi-rotor aerial vehicles. Some of them rely only on observed input-output data; for instance in [1], a frequency domain system identification method was used to obtain a linear representation of the dynamics of a quadrotor. Models that are relatively robust to errors associated with processing noise and bias effects can be obtained from frequency domain methods. On the other hand, in [2], a linear model for the dynamics of a quadrotor was obtained from a time domain system identification technique. In this case, the model structure has to be determined from a large pool of potential candidate regressors. This method is suitable for SISO systems, but characteristics such as cross-coupling are not accounted for. Alternatively, in [3], a black-box model that uses a neural network to learn the dynamics of a quadrotor was proposed. Input-output data can also be used to identify the parameters of general purpose model structures in order to resemble the dynamics of multi-rotor aerial vehicles. For instance, in [4] and [5], an RBF-ARX(Radial Basis Function) model structure was used to represent the dynamics of a quadrotor. The RBF-ARX model is a nonlinear time-varying model with a structure similar to the ARX model. In [6], a Subspace Model Identification (SMI) method was proposed for estimating the state space matrices, of a linear time-invariant continuous-time system, from available data.

Clearly, nonlinear full-coupled dynamic models can be also derived directly from physical principles using formalisms like the Newton-Euler, the Euler-Lagrange or the Quaternion-based approach [7]. Several examples can be found in the literature for modeling the multi-rotor dynamics using the former approaches: [8,9,10,11,12,13,14,15]. In general, the nonlinear identification problem in order to estimate the model parameters from measured data is a difficult problem that has not been yet treated fully in general [16]. An approach for handling the non-linearities consists of developing local models obtained from linearization around different working points. In this case, linear identification methods can be used for identifying the local linear models [17]. However, the linearization can produce oversimplified linear models that can neglect real dynamics, which, for several applications, are important to take into account (e.g., aggressive maneuvers).

If a nonlinear model is used for representing the dynamics of a specific multi-rotor platform, then it is necessary to identify the model parameters experimentally. For instance, in [8], the inertia parameters of a quadrotor were identified by means of a rotational pendulum, and the rotor parameters were statically identified using a test bed. In [11], an Unscented Kalman Filter (UKF) was used for estimating the inertia parameters. In [14], the thrust and rotor drag coefficient were statically identified using a test bed. The work in [15] presented the development of a test bed that can be used for identifying the rotor parameters. In this latter work, the inertia parameters were identified using a method similar to [8]. Furthermore, the inertia parameters can be obtained from a CAD model [18,19] by modeling all the parts of the multi-rotor using commercial software, such as $SolidWorks^{®}$.

### 1.1. Objectives and Contributions

As can be seen from the previous examples, and to our knowledge, all the approaches that can be found in the literature treat the identification of inertia parameters in a separate experimental process from the rotor (thrust and drag coefficient) parameter identification process. Moreover, model parameters are typically identified in a statical manner using custom-built test beds.

In this present research work, the authors investigate the possibility of estimating all the model parameters by means of a unique close-loop estimation process, from measurements that can be obtained directly from the onboard sensors that are commonly available in any multi-rotor setup. Therefore, the objective is to develop an algorithm that is potentially suitable to be used for online identification of the model parameters, thus avoiding the need for laboratory-based identification experiments that make use of custom-built or commercially-available test beds.

The proposed approach is based on the theoretical results obtained from a nonlinear observability analysis. The objective is to find the theoretical conditions needed for estimating the model parameters from different sets of sensor measurements. If a system is observable, then all its internal states can be estimated in a finite time by measuring the system output. Three classes of multi-rotor aerial vehicles are considered in order to carry out the observability analysis. In this case, the state vector of the aerial vehicle is augmented by considering the parameters to be identified as state variables with zero dynamics. Based on this analysis, the sets of measurements and the necessary conditions that must be satisfied will be derived. Those measurements will allow the estimation of the model parameters. An Extended Kalman Filter (EKF) is used for estimating the model parameters. However, since observability properties are inherent to a system, then another state-space-based estimation technique could also be used for the same purpose. In this sense, it is important to note that EKF has been also previously used for parameter estimation of aerial vehicles. For instance, in [20], the aerodynamic parameters of a fixed-wing aircraft and a rotary-wing (helicopter) UAV were estimated. More recently, in [21], a linear Kalman filter-based method was proposed for identifying the dynamic model parameters of quadrotors using flight data. The above method is similar to our work in the sense that both approaches aim to estimate the dynamic model parameters using data available from the onboard sensors. However, in [21], the parameters to be estimated were grouped into coefficients. In our work, the model parameters are explicitly estimated (i.e., inertia parameters). Furthermore, in the previous work, a linear analysis approach was used, while in the present work, a non-linear approach is proposed. Moreover, in [21], only a subset of coefficients was recursively estimated (the least squares method was also used), while in this proposed approach, all the parameters are recursively estimated by means of EKF. Additionally, the proposed approach is developed for three class of multi-rotor vehicles instead of one.

Because the proposed identification method operates in a closed-loop manner and a multi-rotor is open-loop unstable, the identification experiments must be carried out in closed loop, by means of a human operator or automatic control. However, even in the case of custom-built multi-rotors, it is not difficult to use hand-tuned available low-cost autopilots to achieve the maneuvers needed for the identification process. While the proposed technique is suitable for on-line identification, in this work, and for the sake of simplicity, it will be assumed that the data are collected in a flight log during a manually-controlled flight to be processed off-line afterwards.

### 1.2. Paper Outline

The document is structured as follows: Section 2 presents the 12-state model used for representing the kinematics and dynamics of a multi-rotor vehicle. Section 3 presents the specifications and assumptions for the proposed system. Section 4 presents the nonlinear observability analysis from which the theoretical bases of the proposed method are obtained. Section 5 presents the EKF-based scheme used for estimating the model parameters. In Section 6, the results obtained from numerical simulations are shown in order to validate the proposal. Finally, in Section 7, some concluding remarks are given.

## 2. Multi-Rotor Modeling

**Figure 1:**
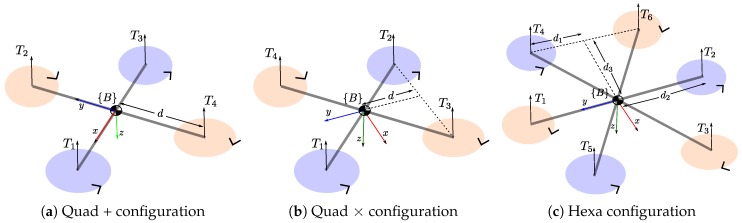
Notation for three common classes of multi-rotor UAV showing their rotors, thrust vectors, and direction of rotation.

In this section, a mathematical model of a multi-rotor UAV is presented. For the analysis presented in this work, three common classes of multi-rotor vehicles will be considered: (i) a quadrotor in + configuration, (ii) a quadrotor in × configuration, and (iii) a hexarotor. Furthermore, note that it should be straightforward to extend the proposed approach to other multi-rotor UAV configurations. Figure 1 shows the notation used for these three common classes of multi-rotor UAV.

In a multi-rotor UAV, the thrust $T_{i}=b\omega_{i}^{2}${i=1,2,3,..,n} is an upward vector generated by each rotor driven by an electric motor. $\omega_{i}$ is the rotor speed, and b>0 is the lift constant that depends on the air density and the characteristics of the blade. $T=\sum T_{i}$ is the total upward thrust. An aerodynamic drag $Q_{i}=k\omega_{i}^{2}$ is opposed to the torque applied to each propeller by the motor. Pairwise differences in rotor thrust cause the vehicle to rotate. For instant, for the quadrotor in × configuration described in Figure 1b, the torque about the *x*-axis (rolling torque) is $\tau_{x}=dT_{2}+dT_{3}-dT_{1}-dT_{4}$, where *d* is the distance from center of mass to the rotors generating the thrust.

The total thrust *T* and torques $\left(\tau_{x},\tau_{y},\tau_{z}\right)$ produced by varying each rotor speed $\omega_{i}$ can be defined in a compact way using a matrix equation. For the case of the quadrotor in + configuration (see Figure 1a):

(1) $$\left[\begin{array}{c} T\\ \tau_{x}\\ \tau_{y}\\ \tau_{z} \end{array}\right]=\left[\begin{array}{cccc} b & b & b & b\\ 0 & -db & 0 & db\\ db & 0 & -db & 0\\ k & -k & k & -k \end{array}\right]\left[\begin{array}{c} \omega_{1}^{2}\\ \omega_{2}^{2}\\ \omega_{3}^{2}\\ \omega_{4}^{2} \end{array}\right]$$

For the case of the quadrotor in × configuration (see Figure 1b):

(2) $$\left[\begin{array}{c} T\\ \tau_{x}\\ \tau_{y}\\ \tau_{z} \end{array}\right]=\left[\begin{array}{cccc} b & b & b & b\\ -db & db & db & -db\\ db & -db & db & -db\\ k & k & -k & -k \end{array}\right]\left[\begin{array}{c} \omega_{1}^{2}\\ \omega_{2}^{2}\\ \omega_{3}^{2}\\ \omega_{4}^{2} \end{array}\right]$$

For the case of the hexarotor (see Figure 1c):

(3) $$\left[\begin{array}{c} T\\ \tau_{x}\\ \tau_{y}\\ \tau_{z} \end{array}\right]=\left[\begin{array}{cccccc} b & b & b & b & b & b\\ -d_{2}b & d_{2}b & d_{1}b & -d_{1}b & -d_{1}b & d_{1}b\\ 0 & 0 & d_{3}b & -d_{3}b & d_{3}b & -d_{3}b\\ -k & k & -k & k & k & -k \end{array}\right]\left[\begin{array}{c} \omega_{1}^{2}\\ \omega_{2}^{2}\\ \omega_{3}^{2}\\ \omega_{4}^{2}\\ \omega_{5}^{2}\\ \omega_{6}^{2} \end{array}\right]$$

The following state vector will be used for representing the motion of the multi-rotor:

(4) $$x_{v}={\left[\begin{array}{cccccccccccc} p_{n} & p_{e} & p_{d} & u & v & w & \phi & \theta & \psi & p & q & r \end{array}\right]}^{T}$$

The north-east-down position $\left(p_{n},p_{e},p_{d}\right)$ is defined relative to the inertial frame ${}^{W}$. The linear velocities (u,v,w) and the angular velocities (p,q,r) are defined relative to the body frame ${}^{B}$. The Euler angles, roll ϕ, pitch θ, and heading (yaw) ψ represent the orientation of the vehicle.

A six-degree-of-freedom, 12-state model for the UAV kinematics and dynamics is given by the following equations [22]:

(5) $$\left[\begin{array}{c} \dot{p_{n}}\\ \dot{p_{e}}\\ \dot{p_{d}} \end{array}\right]=R^{BW}\left[\begin{array}{c} u\\ v\\ w \end{array}\right]$$

(6) $$\left[\begin{array}{c} \dot{u}\\ \dot{v}\\ \dot{w} \end{array}\right]=\left[\begin{array}{c} rv-qw\\ pw-ru\\ qu-pv \end{array}\right]+\frac{1}{m}\left[R^{WB}\left[\begin{array}{c} 0\\ 0\\ mg \end{array}\right]-\left[\begin{array}{c} 0\\ 0\\ T \end{array}\right]\right]$$

(7) $$\left[\begin{array}{c} \dot{\phi }\\ \dot{\theta }\\ \dot{\psi } \end{array}\right]=\left[\begin{array}{ccc} 1 & \sin\phi \tan\theta & \cos\phi \tan\theta \\ 0 & \cos\phi & -\sin\phi \\ 0 & \frac{\sin\phi }{\cos\theta } & \frac{\cos\phi }{\cos\theta } \end{array}\right]\left[\begin{array}{c} p\\ q\\ r \end{array}\right]$$

(8) $$\left[\begin{array}{c} \dot{p}\\ \dot{q}\\ \dot{r} \end{array}\right]=J^{-1}\left[-w_{b/i}^{b}\times Jw_{b/i}+\left[\begin{array}{c} \tau_{x}\\ \tau_{y}\\ \tau_{z} \end{array}\right]\right]$$

where:

(9) $$w_{b/i}^{b}=\left[\begin{array}{c} p\\ q\\ r \end{array}\right]\;J=\left[\begin{array}{ccc} J_{x} & 0 & 0\\ 0 & J_{y} & 0\\ 0 & 0 & J_{z} \end{array}\right]$$

where *m* is the mass of the UAV and *g* is the gravitational constant. $R^{WB}={\left(R^{WB}\right)}^{T}$ is the rotation matrix that transforms from the inertial frame ${}^{W}$ to the body frame ${}^{B}$. J is the inertia matrix of the vehicle. For multi-rotor vehicles, a common assumption is that the mass distribution is closely symmetrical with respect to the vehicle coordinate frame. In this case, the products of inertia of J are zero, and only the moments of inertia $\left(J_{x},J_{y},J_{z}\right)$ will be considered.

Since *d*, $d_{1}$, $d_{2}$, $d_{3}$, and *m* are parameters that will be easily measured, $J_{x}$, $J_{y}$, $J_{z}$, *b*, and *k* are parameters whose values must be identified in order to define a dynamic model of a specific multi-rotor UAV.

## 3. System Definition

For the proposed approach, the model parameters $\left(J_{x},J_{y},J_{z},b,k\right)$ will be treated as state variables, in order to estimate them from a set of measurements obtained from the sensors commonly available in a multi-rotor UAV. In this case, the state vector of the vehicle $x_{v}$ Equation (4) will be augmented as follows, in order to define the system state x:

(10) $$x={\left[\begin{array}{ccccccccccccccccc} p_{n} & p_{e} & p_{d} & u & v & w & \phi & \theta & \psi & p & q & r & J_{x} & J_{y} & J_{z} & b & k \end{array}\right]}^{T}$$

For the parameters, zero dynamics will be assumed:

(11) $${\dot{J}}_{x}={\dot{J}}_{y}={\dot{J}}_{z}=\dot{b}=\dot{k}=0$$

In practical scenarios, UAVs are commonly equipped with a set of sensors that at least include: an Inertial Measurements Unit (IMU) and a Global Positioning System (GPS). At the same time, IMUs typically include accelerometers, gyroscopes, magnetometers, and barometer. The navigation systems make use of the onboard sensors in order to estimate the state of the vehicle. In this case, the most common approaches for addressing the problem of the UAVs’ navigation (position estimation) is the Inertial Navigation Systems (INS), which typically fuses the GPS measurements and inertial measurements from the IMU. Furthermore, the available Attitude and Heading Reference System (AHRS), which commonly relies on the IMU, can be used to provide a reliable estimation of the attitude of the vehicle. Moreover, it is also common that navigation systems now include visual-based sensors like optical flow sensors or cameras in order to improve position estimation.

**Figure 2:**
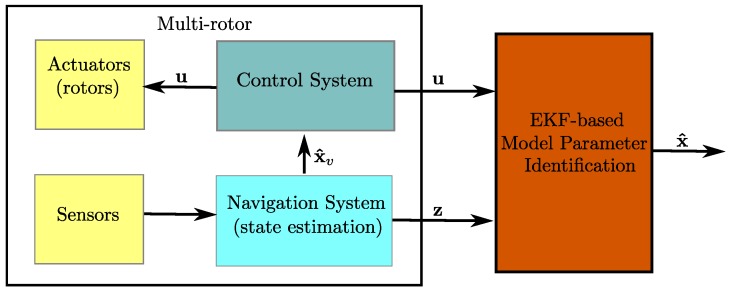
Block diagram of the proposed EKF-based model parameter identification approach.

Figure 2 shows a simplified block diagram illustrating a high-level abstraction of the proposed EKF-based model parameter identification approach. At this stage, for the investigation purposes, it will be assumed that the following measurement vector z can be obtained from the navigation system or directly from the sensors mounted on the multi-rotor:

(12) $$z={\left[\begin{array}{cccccccccccc} p_{n} & p_{e} & p_{d} & u & v & w & \phi & \theta & \psi & p & q & r \end{array}\right]}^{T}$$

Note that the former assumption is equivalent to saying that the whole state of the vehicle Equation (4) is available. However, later, it will be shown that only subsets of z are required in order to estimate the model parameters $\left(J_{x},J_{y},J_{z},b,k\right)$.

It is also important to note that there is no theoretical restriction about the manner that measurements are obtained. For instance, in order to measure z, the angular velocities (p,q,r) can be obtained directly from the IMU; the attitude (ϕ,θ,ψ) can be obtained from the AHRS; and the position $\left(p_{n},p_{e},p_{d}\right)$ and linear velocities (u,v,w) can be obtained from the INS. Of course, other configurations may be possible while the required state measurements are available. Even, an external motion capture system (e.g., VICON or OptiTrack) could be used for the same purpose. However, this work is mainly intended to show that the model parameters can be estimated by using only the onboard sensors commonly available in multi-rotor UAVs.

Additionally, it will be assumed that the control input u, which commands the rotors speed $\omega_{i}$, is available from the control system. In many practical scenarios, the vector u will be composed by the values of PWM (Pulse-Width Modulation) signals that are used as reference inputs of the ESC (Electronic Speed Control) units that control the rotational speed of the rotors. Obviously, if tachometers are available, or if the relation between the PWM signal and the rotor speed is known, then the vector u can be composed by the rotor speed (i.e., $u=[\omega_{1},\omega_{2},..,\omega_{n}]$).

## 4. Observability Analysis

The observability properties of the nonlinear system defined by Equations (5)–(8) and (11) will be studied by means of a nonlinear observability analysis. The observability is a measure about how well internal states of a system can be inferred from knowledge of their external outputs (measurements). The objective is to find the theoretical conditions needed for estimating the state vector x Equation (10) and in particular the state variables $\left(J_{x},J_{y},J_{z},b,k\right)$ from the measured vector z Equation (12) or a subset of it.

A system is defined as observable if the initial state $x_{0}$, at any initial time $t_{0}$, can be determined given the state transition model of the system $\dot{x}=f\left(x\right)$, the measurement model of the system y=h(x), and the observations $z[t_{0},t]$, from time $t_{0}$ to a finite time *t*. In [23], it was proven that a non-linear system is *locally weakly observable* if the observability rank condition rank(O)=dim(x) is verified, where O is the observability matrix. Matrix O can be computed from:

(13) $$O={\left[\begin{array}{ccc} \frac{\partial (L_{f}^{0}h_{1})}{\partial x}\frac{\partial (L_{f}^{1}h_{1})}{\partial x}..\frac{\partial (L_{f}^{n}h_{1})}{\partial x} & \frac{\partial (L_{f}^{0}h_{2})}{\partial x}\frac{\partial (L_{f}^{1}h_{2})}{\partial x}..\frac{\partial (L_{f}^{n}h_{2})}{\partial x}... & \frac{\partial (L_{f}^{0}h_{m})}{\partial x}\frac{\partial (L_{f}^{1}h_{m})}{\partial x}..\frac{\partial (L_{f}^{n}h_{m})}{\partial x} \end{array}\right]}^{T}$$

where $L_{f}^{n}h_{m}$ is the *nth*-order Lie derivative [24], of the *mth* scalar field $h_{m}$ with respect to the vector field f.

Independent of the sensory source from which each component of the measurement vector z is obtained (see Section 3), it will be assumed that the vector z can be predicted directly from the state vector x. In this case, the measurement models $h_{i}\left(x\right)$, where *i* is the index for the predicted component of z, will simply be defined as follows:

(14) $$h_{1}\left(x\right)=p_{n}\;h_{2}\left(x\right)=p_{e}\;h_{3}\left(x\right)=p_{d}\;h_{4}\left(x\right)=u\;...\;h_{12}\left(x\right)=r$$

There are no rules regarding the order of Lie derivatives used for constructing the observability matrix O. In this work, only {0,1,2}-order Lie derivatives are tested for each measurement model $h_{i}\left(x\right)$.

The observability matrices were computed numerically by means of the symbolic $MATLAB^{®}$ toolbox. Table 1 shows the sets of measurements from which the full rank matrix condition (rank(O)=dim(x)) is accomplished. That means that if the state vector $x_{v}$ is available to be measured, then the parameters $\left(J_{x},J_{y},J_{z},b,k\right)$ can be indirectly estimated when treated as state variables (see Configuration **(a)**). Moreover, this means that the state vector x Equation (10), and thus the model parameters, can be observable even if the linear velocities (u,v,w) are not measured (see Configuration **(b)**).

Table 2 shows the sets of measurements from which the full rank matrix condition (rank(O)=dim(x)) is not accomplished, but from which the unobservable modes are related only to the vehicle state variables of $x_{v}$, meaning that the model parameters $\left(J_{x},J_{y},J_{z},b,k\right)$ are still observable. That means that for instance, and at least theoretically, the model parameters can be estimated using only inertial measurements (see Configuration **(e)**). Moreover, the minimal set of sensors needed for estimating the model parameters appears to be the Configuration **(f)**. In this case, only angular measurements and measurement of the vertical position $p_{d}$ are considered.

Observing Table 1 and Table 2, it can be seen that the measurement of angular velocities (p,q,r) represents a necessary condition for the observability of the model parameters. Furthermore, in order to obtain the above observability results, a necessary condition that must be satisfied is p≠0 and q≠0. That means that the aerial vehicle must be varying its attitude during the data capture. The same observability results were found for the three multi-rotor configurations shown in Figure 1.

## 5. Kalman Filtering

In Section 4, the observability properties of the multi-rotor UAV model were investigated. From that analysis, some conditions were found that theoretically should allow estimating the multi-rotor model parameters from data obtained with the on-board sensors. In this work, an Extended Kalman Filter (EKF) is used as the estimation technique for this purpose. On the other hand, it is important to note that the observability properties are inherent to a system, and therefore are independent of the estimation technique. Therefore, the theoretical results presented in this work should also be valid for implementing another state-space-based estimation techniques (e.g., particle filtering, unscented Kalman filtering, etc.) for identifying the model parameters.

In order to apply the EKF, and since the sensor data were obtained in a discrete manner, a discrete-stochastic system model is characterized by the continuous dynamics $\dot{x}=f\left(x,u\right)$ defined by Equations (5)–(8) and (11). In this work, the Euler method is used for this purpose, and therefore:

(15) $$x_{k}=f\left(x_{k-1},u_{k-1},n_{k-1}\right)=\left(x_{k-1}+f\left(x_{k-1},u_{k-1}\right)\Delta t\right)+n_{k}$$

The system measurement prediction model is defined by:

(16) $$h\left(x_{k},r_{k}\right)={\left[\begin{array}{cccc} h_{1}\left(x_{k}\right) & h_{2}\left(x_{k}\right) & ... & h_{n}\left(x_{k}\right) \end{array}\right]}^{T}+r_{k}$$

Let $n_{k}∼N\left(0,Q_{k}\right)$ and $r_{k}∼N\left(0,R_{k}\right)$ be the noise vectors that affect the state and the measurements, which are assumed to be mutually uncorrelated. Let Δt be the differential of time and *k* the sample step.

The prediction equations of the EKF are:

(17) $${\hat{x}}_{k}^{-}=f\left({\hat{x}}_{k-1},u_{k-1},0\right)$$

(18) $$P_{k}^{-}=A_{k}P_{k-1}A_{k}^{T}+Q_{k-1}$$

The update equations of the EKF are:

(19) $${\hat{x}}_{k}={\hat{x}}_{k}^{-}+K_{k}\left(z_{k}-h\left({\hat{x}}_{k}^{-},0\right)\right)$$

(20) $$P_{k}=\left(I-K_{k}C_{k}\right)P_{k}^{-}$$

with:

(21) $$K_{k}=P_{k}^{-}C_{k}^{T}{\left(C_{k}P_{k}^{-}C_{k}^{T}+R_{k}\right)}^{-1}$$

and:

(22) $$\begin{array}{c} A_{k}=\frac{\partial f}{\partial x}\left({\hat{x}}_{k-1},u_{k-1},0\right)\;C_{k}=\frac{\partial h}{\partial x}\left({\hat{x}}_{k}^{-},0\right) \end{array}$$

Let $z_{k}$ be the vector of actual measurements at step *k*, P be the covariance matrix of the system state, and K be the Kalman gain.

Let $A_{k}$ be the Jacobian defined by the derivatives $\frac{\partial f}{\partial x}$ of the prediction model f with respect to the system state x. Let $C_{k}$ be the Jacobian defined by the derivatives $\frac{\partial h}{\partial x}$ of the prediction model h with respect to the system state x. For the sake of simplicity, the expressions for derivatives are not included.

**Figure 3:**
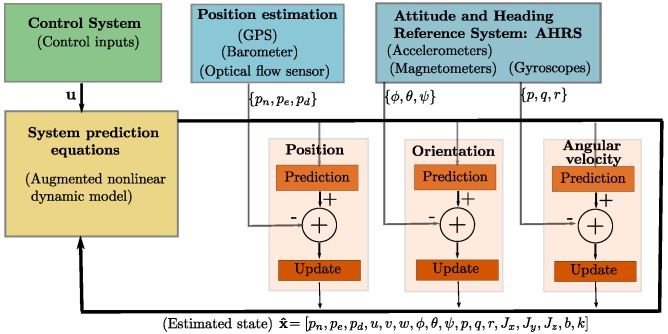
Diagram of the EKF in Configuration **(b)**.

The update Equations (19)–(21) are used according to the available set of measurements. For instance, Figure 3 shows a diagram illustrating the data flow for the EKF in/Configuration **(b)**. In this case, the linear velocities (u,v,w) are not used for updating the system state $\hat{x}$ (see Section 4). It is well worth noting that, as indicated in Figure 3, the filter was updated in a separate manner just after each type of measurement is available.

## 6. Results

In this section, experimental results are presented in order to show the performance of the proposed method. In this case, computer simulations were carried out to validate in a numerical way the proposed approach and the theoretical findings. Furthermore, experiments with real data obtained from a quadrotor were performed to have a better insight into the performance of the proposed method.

### 6.1. Simulations

In order to test the proposed EKF-based model parameter identification approach, under all the observability configurations defined in Section 4, a multi-rotor system as described in Figure 2 was simulated using $Simulink^{®}$ from $MATLAB^{®}$. In this case, since all observability results were the same for the three multi-rotor configurations investigated, only one configuration was tested: a quadrotor in × configuration (Figure 1b). Table 3 shows the actual parameter values of the simulated quadrotor.

To implement the control system, a hand-tuned altitude and orientation PI control scheme was used. In this case, the control reference signals were: $\left(p_{d_{r}},\phi_{r},\theta_{r},\psi_{r}\right)$. Note that by controlling only these four variables, the remaining states can also be excited. The control vector u was obtained from the control system.

**Figure 4:**
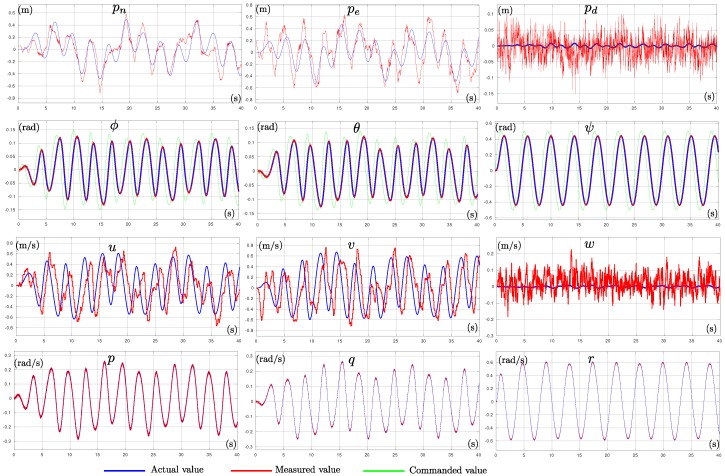
Control reference signals (green). Actual quadrotor states (blue). Measured quadrotor states (red).

In order to obtain the measurement vector z Equation (12), the following sensors scheme was used. Angular velocities (p,q,r) were obtained by emulating the noise specifications of an Invensense MPU-6000 inertial measurement unit. The attitude (ϕ,θ,ψ) of the multi-rotor was provided by an AHRS, as described in [25], which in turn used the same IMU measurements. Position measurements $\left(p_{n},p_{e},p_{d}\right)$ were obtained from smoothing GPS measurements with a linear Kalman filter. For the altitude measurement $\left(p_{d}\right)$, the filter also fused measurements obtained from an altitude sensor configured to emulate the noise specifications of the Adafruit BMP183 barometric low-cost sensor. To model the transient behavior of the GPS error, the approach of [26] was followed. This Kalman filter also included, as state variables, the derivatives of the position variables, in order to provide the linear velocities (u,v,w) of the vehicle. Figure 4 shows a comparison between actual states of the quadrotor and the measured states used for estimating its model parameters. In this case, note that for instance the position and linear velocities measurements were not very precise. Table 4 shows the diagonal values of the matrices $P_{ini}$ (initial values), *Q*, and *R* used in simulations.

#### 6.1.1. Sensors Configurations Test

All the sensors configurations **(a**–**f)**, from whose parameters $\left(J_{x},J_{y},J_{z},b,k\right)$ were observable (see Section 4), were tested. Figure 4 shows the control reference signals $\left(p_{d_{r}},\phi_{r},\theta_{r},\psi_{r}\right)$ used for exciting the quadrotor state (green signals). Note that the quadrotor was commanded to maintain a stable altitude $p_{d}$, while its orientation (ϕ,θ,ψ) was excited by sinusoidal signals. Furthermore, the actual values for all the vehicle states in $x_{v}$ are shown (blue signals). The measured vehicle states, used for estimating the model parameters, are illustrated by the red signals.

**Figure 5:**
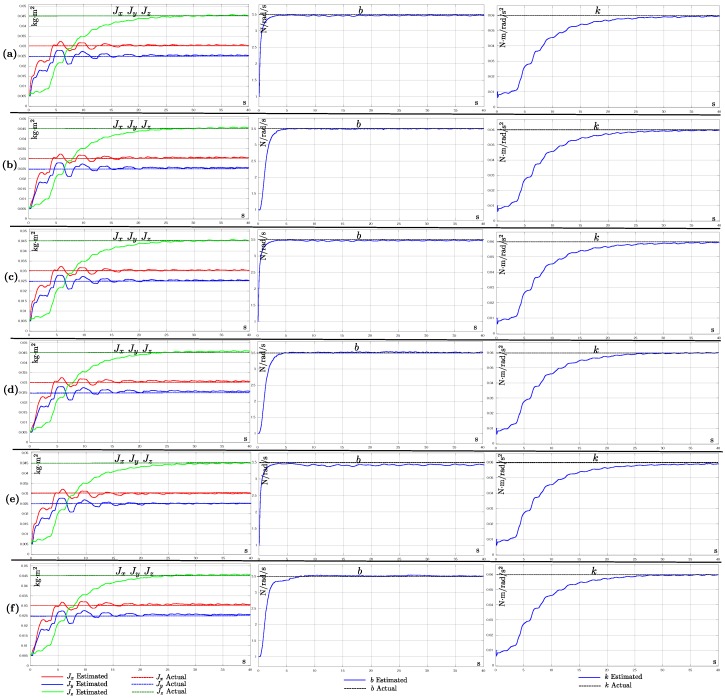
Estimates of the model parameters values $\left(J_{x},J_{y},J_{z},b,k\right)$ obtained from each sensor configurations (**a**–**f**). Each row of plots shows the results of a different sensor configuration.

Figure 5 shows the evolution over time of the estimated model parameter values obtained from each sensor configuration **(a**–**f)**. For instance, in this case, the first row of plots (Configuration **(a)**) shows the results obtained when the measurements of all the vehicle states are used for updating the EKF. In the same manner, the last row of plots (configuration **(f)**) shows the results obtained when only measurements of altitude and angular velocities were used for updating the filter. Observing Figure 5, it can be seen that, with all the sensors configurations, it was possible to estimate in a good manner the model parameters values $\left(J_{x},J_{y},J_{z},b,k\right)$. Table 5 shows the average values of the parameters estimated and their respective mean errors obtained for each sensor configuration. The above results validated the theoretical findings obtained from the observability analysis regarding the measurements needed for estimating the parameters.

#### 6.1.2. Observability Conditions Test

From the observability results (Section 4), it was found that a necessary condition, which must be satisfied in order to make observable the full set of model parameters, had to fulfill that p≠0 and q≠0. This result had important implications for the success of the method because it implied that the aerial vehicle must be varying its attitude in order to estimate the parameters’ values. In order to validate the observability necessary conditions, two tests were carried out. For these tests, only the results obtained from the sensors in Configuration **(d)** are shown, but similar results were obtained with the other configurations. The following cases were tested:

(i) *Case 1*. For the first case, the quadrotor was commanded to ascend and maintain a stable altitude (hovering position). Therefore, from time *t* = 15 s to the end of the simulation, the yaw angle ψ of the quadrotor was varied.

(ii) *Case 2*. For the second case, the quadrotor was commanded to ascend and maintain a stable altitude (hovering position). Then, from time *t* = 10 s to the end of the simulation, the roll angle ϕ of the quadrotor was varied. From time *t* = 25 s to the end of the simulation, the pitch angle θ of the quadrotor was varied. Finally, from time *t* = 40 s to the end of the simulation, the yaw angle ψ of the quadrotor was varied.

**Figure 6:**
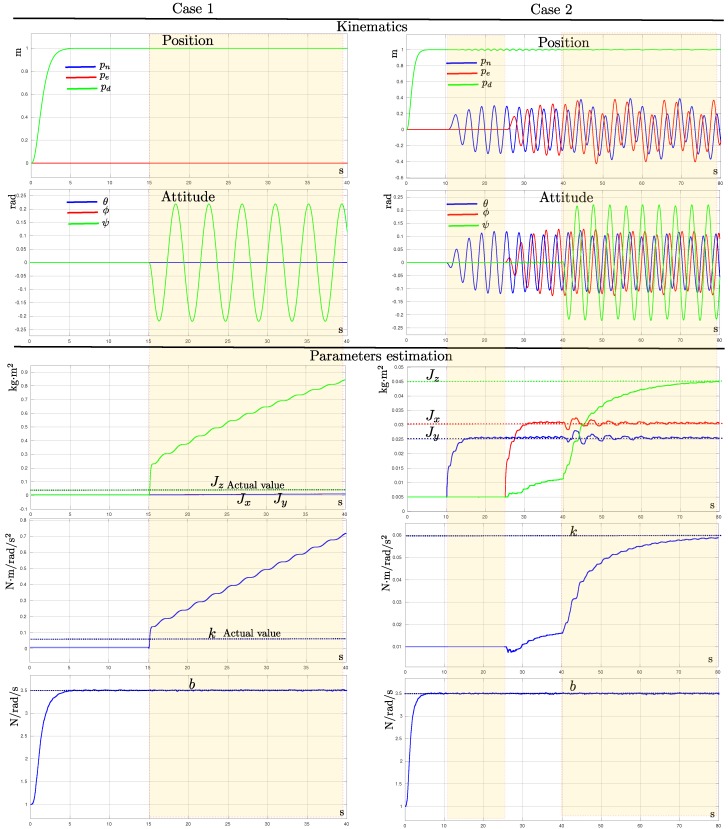
Parameter estimates obtained from different observability conditions. The left and right plots show respectively the results obtained in the *Case 1* and *Case 2* tests. The upper plots show the maneuvers of the aerial vehicle carried out to establish the different observability conditions. The lower plots show the parameter estimates.

Figure 6 shows the results obtained *Case 1* (left column plots) and *Case 2* (right column plots). The upper two rows of plots show the movements (position and attitude) of the quadrotor for each case. The lower plots show the evolution over time of the values estimated for each model parameter. Analyzing the plots, the following remarks can be stated. The estimation of parameter *b* did not depend on the movement of the multi-rotor vehicle; even if the aerial vehicle was maintained in a hover position. Observing *Case 1*, it was clear that only moving the multi-rotor vehicle over its *z*-axis was not enough to estimate the parameters *k* and $J_{z}$. In this case, a variation in the yaw ψ excited the states of these parameters (*k* and $J_{z}$), but their estimates clearly diverged from the actual values. Observing *Case 2*, it can be observed that just moving the multi-rotor over its *x*-axis (roll ψ), the parameter $J_{x}$ became observable, but not the parameters $J_{z}$, $J_{y}$, and *k*. It is not illustrated in the plots, but the same effect occurred if the multi-rotor was rotated only over its *y*-axis (in addition to *b*, only the parameter $J_{y}$ was observable). On the other hand, the fact of accomplishing the sufficient conditions (p≠0 and q≠0) made all the parameters observable. In this case, it is important to note that varying roll and pitch angles produced a small perturbation over the yaw of the vehicle (see t the plots from time *t* = 25 s to *t* = 40 s), thus exciting the states of the related *z*-axis parameters (i.e., *k* and $J_{z}$). However, in the above case, the convergence was very slow. Note that, when the yaw ψ of the multi-rotor vehicle varied abruptly (which happened in *t* = 40 s), the convergence of parameters *k* and $J_{z}$ became faster. The results obtained from these simulation tests validated the theoretical findings derived from the observability analysis.

### 6.2. Experimental Case

**Figure 7:**
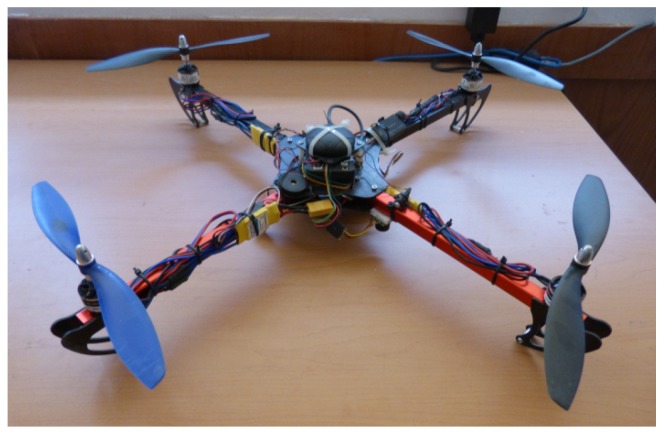
Quadrotor in × configuration whose model parameters were estimated using the proposed method.

In order to validate experimentally the proposed method with real data, the model parameters of a custom-built quadrotor in × configuration were estimated (see Figure 7). The quadrotor was equipped with a standard Pixhawk-PX4 autopilot unit. The on-board sensors included in the Pixhawk were: Accel/Gyro ICM-20689, Accel/Gyro BMI055, Magnetometer IST8310, and Barometer MS5611.

**Figure 8:**
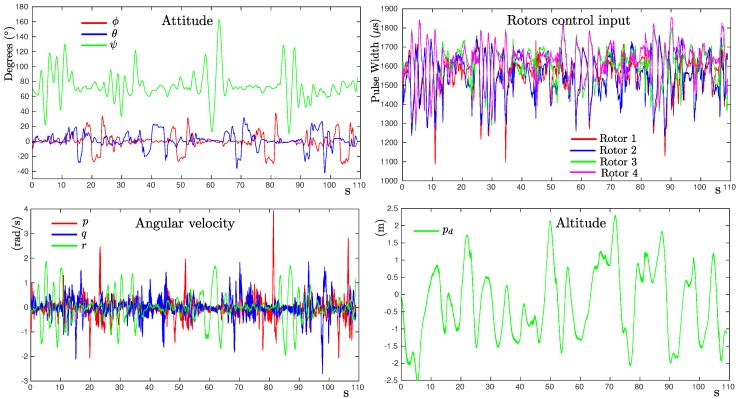
Flight log data used to estimate the model parameters $\left(J_{x},J_{y},J_{z},b,k\right)$.

For performing the experiments, the quadrotor was flown manually by means of a radio transmitter. In order to accomplish the observability conditions, during the flights, the quadrotor was commanded to vary as much as possible its attitude. During each flight, the Pixhawk-PX4 created a log file in which the sensors’ data and other several data, like the attitude, position, and velocities estimates, were stored. Therefore, for estimating the model parameters of the quadrotor, the flight log data were used as input to a $MATLAB^{®}$ implementation of the proposed method. In this case, the following data were used: (i) raw angular velocity from gyros {p,q,r}, (ii) estimated attitude {ϕ,θ,ψ}, (iii) estimated altitude $\left\{p_{d}\right\}$, and (iv) PWM signals used for controlling the rotors speed, (see Figure 8).

**Figure 9:**
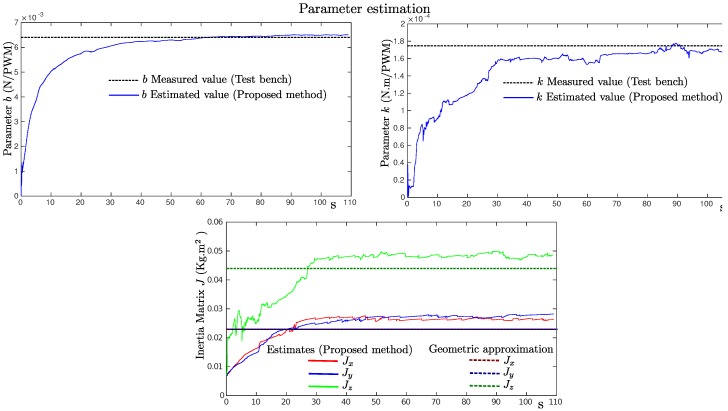
Estimates of the model parameters $\left(J_{x},J_{y},J_{z},b,k\right)$ obtained from the flight log data of the quadrotor.

**Figure 10:**
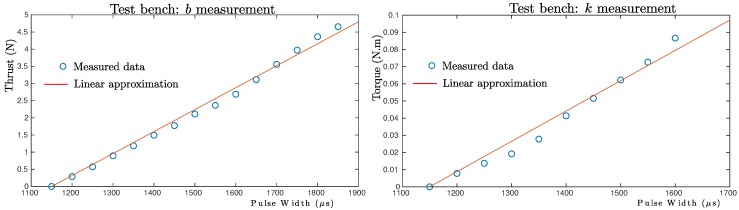
Linear approximation models obtained for the thrust coefficient *b* and the drag coefficient *k* using a custom-built test bench.

Figure 9 shows the evolution over time of the estimated model parameter values of the quadrotor. For comparison purposes, the thrust coefficient *b* and the drag coefficient *k* were measured using a custom-built test bench in a similar manner as in [14]. Figure 10 shows the measured data and the linear approximation that best fit the data. According to the linear approximation, the following values were measured: *b* = 0.0064 (N/PWM) and *k* = 1.766 ×$10^{4}$ (N·m/PWM). Inertia matrix parameters ($J_{x}$,$J_{y}$,$J_{z}$) were experimentally measured using a test bench (see [8]). Therefore, in order to have at least an insight about the values of these parameters, a geometric approximation method as that proposed in [27] was used. Observing Figure 9 (lower plot), a small offset between the estimated values and the values obtained from the geometric approximation can be seen.

## 7. Conclusions

In this work, a method for identifying the model parameters of a multi-rotor vehicle was presented, using a single online EKF-based estimation process that integrated measurements that can be obtained directly from onboard sensors commonly available in this class of UAVs. The objective was to provide a practical and reliable estimate of the model parameters, but without the need for static test-bed-based identification experiments.

In order to investigate the theoretical conditions that were necessary for estimating the model parameters from different sets of sensor measurements, a nonlinear observability analysis was carried out. As an outcome of this analysis, several sensor configurations along with sufficient conditions of observability were obtained. In this work, an extended Kalman filter was used as the estimation technique for identifying the model parameters. However, in future works, it could be of interest to test other state-space-based estimation techniques, since observability properties are inherent to a system, and they do not depend on a particular estimation technique. Since the proposed method worked in a closed-loop manner, the requirement of operation was by means of a human operator or automatic control of the multi-rotor vehicle that carried out the flight maneuvers needed for accomplishing the observability conditions of the system.

In order to validate the proposed method, an extensive set of computer simulations was carried out. The results obtained from simulation supported the theoretical findings. Hence, it was feasible to estimate all the model parameters in a single estimation process from measurements that could be obtained directly from the onboard sensors of the multi-rotor. Moreover, the model parameters of a custom-built quadrotor were estimated from actual flight log data. The experimental results showed that the proposed method could also be applied in a practical manner.

## Figures and Tables

**Table 1:** Sets of measurements from which the full rank matrix condition (rank(O)=dim(x)) is accomplished.

Configuration	$\left\{p_{n},p_{e},p_{d}\right\}$	{u,v,w}	{ϕ,θ,ψ}	{p,q,r}
**(a)**	✓	✓	✓	✓
**(b)**	✓	✗	✓	✓

**Table 2:** Sets of measurements from which the full rank matrix condition (rank(O)=dim(x)) is not accomplished, but from which the parameters $\left(J_{x},J_{y},J_{z},b,k\right)$ can be estimated.

Configuration	$\left\{p_{n},p_{e}\right\}$	$\left\{p_{d}\right\}$	{u,v,w}	{ϕ,θ,ψ}	{p,q,r}
**(c)**	✗	✗	✓	✓	✓
**(d)**	✓	✓	✗	✗	✓
**(e)**	✗	✗	✓	✗	✓
**(f)**	✗	✓	✗	✗	✓

**Table 3:** Actual parameter values.

Parameter	Value	Units	Known
*m*	0.65	kg	✓
*d*	0.165	m	✓
$J_{x}$	0.03	kg·m${}^{2}$	✗
$J_{y}$	0.025	kg·m${}^{2}$	✗
$J_{z}$	0.045	kg·m${}^{2}$	✗
*b*	3.50	N/rad/s	✗
*k*	0.06	N·m/rad/s	✗

**Table 4:** EKF parameter values used in simulations.

Parameter	$p_{n}$	$p_{e}$	$p_{d}$	*u*	*v*	*w*	ϕ	θ	ψ	*p*	*q*	*r*	$J_{x}$	$J_{y}$	$J_{z}$	*b*	*k*
$P_{ini}$	0.1	0.1	0.1	0.1	0.1	0.1	0.1	0.1	0.1	0.1	0.1	0.1	0.001	0.001	0.001	0.01	0.01
*Q*	0.01	0.01	0.01	0.01	0.01	0.01	0.01	0.01	0.01	0.01	0.01	0.01	0.0001	0.0001	0.0001	0.001	0.0001
*R*	0.01	0.01	0.01	0.0025	0.0025	0.0025	0.0025	0.0025	0.0025	0.0025	0.0025	0.0025	-	-	-	-	-

**Table 5:** Average value estimates and mean errors.

Configuration	*b*	$e_{b}$	*k*	$e_{k}$	$J_{x}$	$e_{J_{x}}$	$J_{y}$	$e_{J_{y}}$	$J_{z}$	$e_{J_{z}}$
**(a)**	3.477	2.2 × $10^{-2}$	0.0589	1.0 × $10^{-3}$	0.0304	4.2 × $10^{-4}$	0.0254	4.1 × $10^{-4}$	0.0450	1.2 × $10^{-5}$
**(b)**	3.501	1.6 × $10^{-3}$	0.0592	7.4 × $10^{-4}$	0.0306	1.0 × $10^{-2}$	0.0255	5.8 × $10^{-4}$	0.0452	2.8 × $10^{-4}$
**(c)**	3.474	2.5 × $10^{-2}$	0.0589	1.0 × $10^{-3}$	0.0303	3.9 × $10^{-4}$	0.0254	4.0 × $10^{-4}$	0.0450	3.3 × $10^{-5}$
**(d)**	3.516	1.6 × $10^{-2}$	0.0596	3.9 × $10^{-4}$	0.0307	7.4 × $10^{-4}$	0.0256	6.9 × $10^{-4}$	0.0455	5.5 × $10^{-4}$
**(e)**	3.475	2.4 × $10^{-2}$	0.0590	9.3 × $10^{-4}$	0.0304	4.3 × $10^{-4}$	0.0254	4.3 × $10^{-4}$	0.0451	1.4 × $10^{-4}$
**(f)**	3.509	4.0 × $10^{-2}$	0.0594	8.1 × $10^{-4}$	0.0306	6.9 × $10^{-4}$	0.0256	6.4 × $10^{-4}$	0.0454	6.1 × $10^{-4}$
